# Effects of swift heavy ion irradiation on structural, optical and photocatalytic properties of ZnO–CuO nanocomposites prepared by carbothermal evaporation method

**DOI:** 10.3762/bjnano.6.96

**Published:** 2015-04-10

**Authors:** Sini Kuriakose, D K Avasthi, Satyabrata Mohapatra

**Affiliations:** 1 School of Basic and Applied Sciences, Guru Gobind Singh Indraprastha University, Dwarka, New Delhi 110078, India; 2 Inter University Accelerator Centre, New Delhi 110067, India

**Keywords:** nanocomposites, nanosheets, ion irradiation, photocatalysis, ZnO–CuO

## Abstract

ZnO–CuO nanocomposite thin films were prepared by carbothermal evaporation of ZnO and Cu, combined with annealing. The effects of 90 MeV Ni^7+^ ion irradiation on the structural and optical properties of ZnO–CuO nanocomposites were studied by using X-ray diffraction (XRD), field emission scanning electron microscopy (FESEM), UV–visible absorption spectroscopy and Raman spectroscopy. XRD studies showed the presence of ZnO and CuO nanostructures in the nanocomposites. FESEM images revealed the presence of nanosheets and nanorods in the nanocomposites. The photocatalytic activity of ZnO–CuO nanocomposites was evaluated on the basis of degradation of methylene blue (MB) and methyl orange (MO) dyes under sun light irradiation and it was observed that swift heavy ion irradiation results in significant enhancement in the photocatalytic efficiency of ZnO–CuO nanocomposites towards degradation of MB and MO dyes. The possible mechanism for the enhanced photocatalytic activity of ZnO–CuO nanocomposites is proposed. We attribute the observed enhanced photocatalytic activity of ZnO–CuO nanocomposites to the combined effects of improved sun light utilization and suppression of the recombination of photogenerated charge carriers in ZnO–CuO nanocomposites.

## Introduction

Semiconductor nanocomposites have gained significant attention in the last few decades due to their widespread applications. Various physical and chemical methods such as carbothermal reduction [1], vapour-transport processes [2–3], thermal evaporation [4], wet chemical methods [5] and flame-transport methods [6–7] have been used to fabricate nanocomposites of SnO_2_–ZnO [8], ZnO–ZnS [9], and Zn–ZnO [10]. ZnO, an n-type semiconductor, has attracted significant research interests due to its wide band gap and large exciton binding energy, making it suitable for a wide range of applications such as UV lasers [11], dye-sensitized solar cells [12–14], gas sensors [15–16], UV sensors [17], light emitting diodes [18], spintronic devices [19], transparent conductive electrodes [20], lasers [21], biosensors [22] and photocatalysts [23–25]. Nanocomposites consisting of nanostructures of ZnO and other metal-oxide semiconductors are being widely studied due to their improved physicochemical properties as compared to the individual counterparts. CuO, a p-type narrow band gap semiconductor, is promising for applications ranging from solar cells to lithium-ion batteries [26–29]. ZnO–CuO nanocomposites formed by combining ZnO and CuO nanostructures are expected to exhibit improved physicochemical properties as compared to pure ZnO and CuO nanostructures, because of the formation of nano-heterojunctions leading to a modification of optical and electronic properties, which finds promising applications in photocatalysis [30], sensors [31] and solar cells [32].

Several methods have been used to modify the optical, electrical and structural properties of nanostructured materials and nanocomposite thin films. Swift heavy ion (SHI) irradiation using high electronic excitation is one of the promising techniques used to controllably engineer the size, shape, crystallinity and hence the physicochemical properties of nanostructured materials and nanocomposites. Energetic ions moving in a solid lose energy in elastic and inelastic scattering with the target nuclei and electrons, respectively. The localized deposition of high energy results in the formation of defects and induces structural transformations in solids. SHI irradiation depositing high energy in electronic excitations can lead to formation of latent tracks along ion path and results in material modifications including growth [33] and elongation [34] of nanoparticles embedded in different insulating matrices. Several attempts have been made to study SHI-irradiation-induced changes in the optical, electrical and structural properties of ZnO. Kumar et al. [35] have irradiated Co doped ZnO thin films, prepared by sol–gel route, with 100 MeV Ni^7+^ ions and studied the modifications in their structural and optical properties. Kumar et al. [36] studied 130 MeV Ni^7+^ irradiation induced morphological and optical changes of zinc aluminum oxide coated over porous silicon substrates. The changes in the structural and optical properties of ZnO thin films due to 100 MeV Au^8+^ irradiation were investigated by Agarwal et al. [37]. Even though there have been several studies on the ion-induced evolution of the structural and optical properties of ZnO nanostructures, not much work has been done on the SHI-induced modifications in ZnO–CuO nanocomposites.

In this paper, we report on the effects of 90 MeV Ni^7+^ ion irradiation on the structural, optical and photocatalytic properties of ZnO–CuO nanocomposites, which were prepared by a simple carbothermal reduction-based vapor deposition method. We have demonstrated that swift heavy ion irradiation can be employed to significantly enhance the sun light driven photocatalytic activity of ZnO–CuO nanocomposites toward the degradation of methylene blue (MB) and methyl orange (MO) dyes in water.

## Results and Discussion

**Figure 1:**
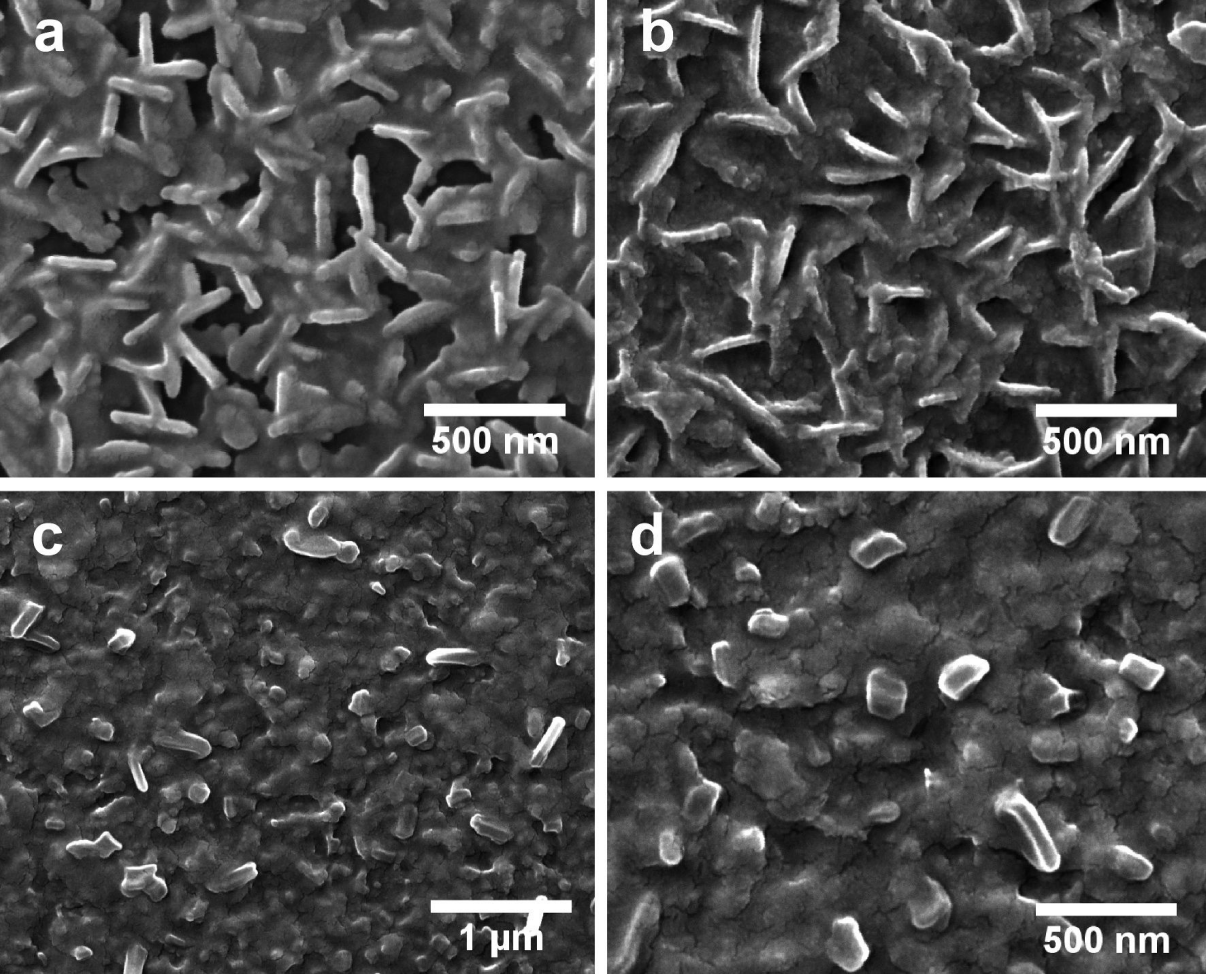
FESEM images of (a) pristine sample, samples irradiated with 90 MeV Ni ions at a fluence of (b) 3 × 10^13^ and (c,d) 1 × 10^14^ ions/cm^2^.

The FESEM images of pristine and irradiated nanocomposite samples are shown in Figure 1. The FESEM image of the pristine sample clearly illustrates the presence of a large number of nanosheets in addition to few nanorod-like structures, as shown in Figure 1a. It can be clearly seen that these nanosheets and nanorod like structures consist of smaller nanoparticles. Figure 1b shows the FESEM image revealing the surface morphology of nanocomposite following irradiation with 90 MeV Ni ions at a fluence of 3 × 10^13^ ions/cm^2^. It can be clearly seen that swift heavy ion irradiation at a fluence of 3 × 10^13^ ions/cm^2^ resulted in the formation of a high density of nanosheets with reduced thickness. The FESEM images showing the surface morphology of nanocomposite following irradiation with 90 MeV Ni ions at a fluence of 1 × 10^14^ ions/cm^2^ are shown in Figure 1c and Figure 1d. The presence of large nanorods with distinct facets and increased width can be clearly seen. However, the density of nanorod-like structures formed is small and the average aspect ratio of such nanostructures was found to be 2.7, which is much smaller than that of the nanostructures in the pristine sample and the samples irradiated at lower fluences. The average thickness of nanosheets decreased from 52 nm (for pristine) to 45 nm at a fluence of 3 × 10^13^ ions/cm^2^, whereas the average thickness of nanostructures increased to 91 nm for the nanocomposite irradiated at a fluence of 1 × 10^14^ ions/cm^2^. The observed change in morphology from nanosheets to nanorods upon swift heavy ion irradiation at higher fluence is really interesting considering the appreciable radiation stability of ZnO.

**Figure 2:**
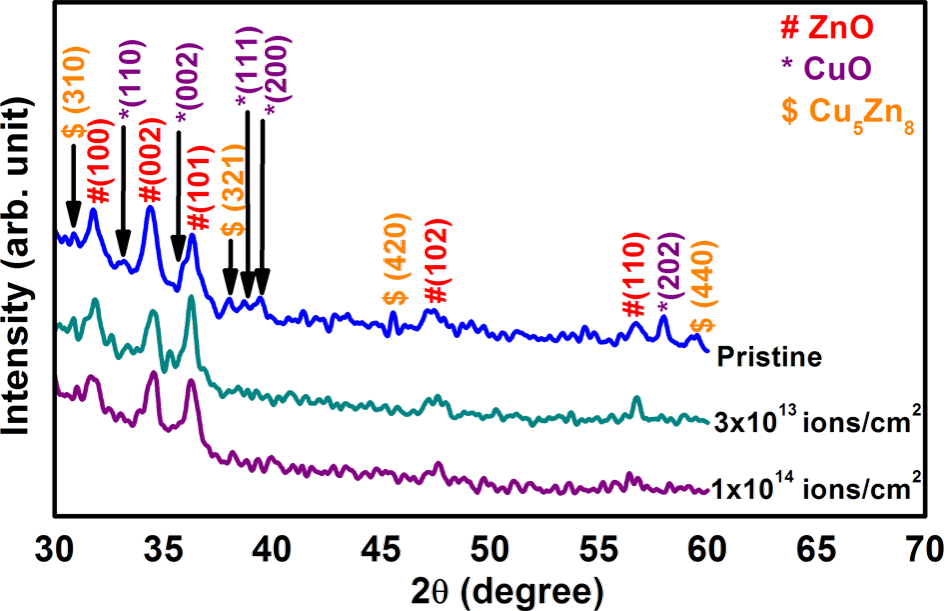
XRD patterns of pristine and irradiated ZnO–CuO nanocomposites.

Figure 2 shows the XRD patterns of pristine nanocomposite and samples irradiated with 90 MeV Ni ions at fluences of 3 × 10^13^ and 1 × 10^14^ ions/cm^2^. All the samples show the peaks for hexagonal wurtzite structure of crystalline ZnO [JCPDS no. 36-1451] and monoclinic structure of CuO [JCPDS no. 80-0076], as marked in the figure. The average crystallite size of ZnO obtained from XRD analysis for the pristine sample and samples irradiated at fluences of 3 × 10^13^ and 1 × 10^14^ ions/cm^2^ are estimated to be 14.2 nm, 14.6 nm and 15.7 nm, respectively. It can be clearly seen that the average crystallite size of ZnO increases with increase in ion fluence. In addition to ZnO and CuO, nanostructured Cu–Zn alloy, Cu_5_Zn_8_ [JCPDS no. 71-0397] were also observed, as can be seen from Figure 2. The Cu–Zn alloy is more prominently seen in the case of pristine sample. In the swift heavy ion irradiated samples the peaks corresponding to Cu–Zn alloy nanoparticles get increasingly diminished in intensity with increase in fluence.

**Figure 3:**
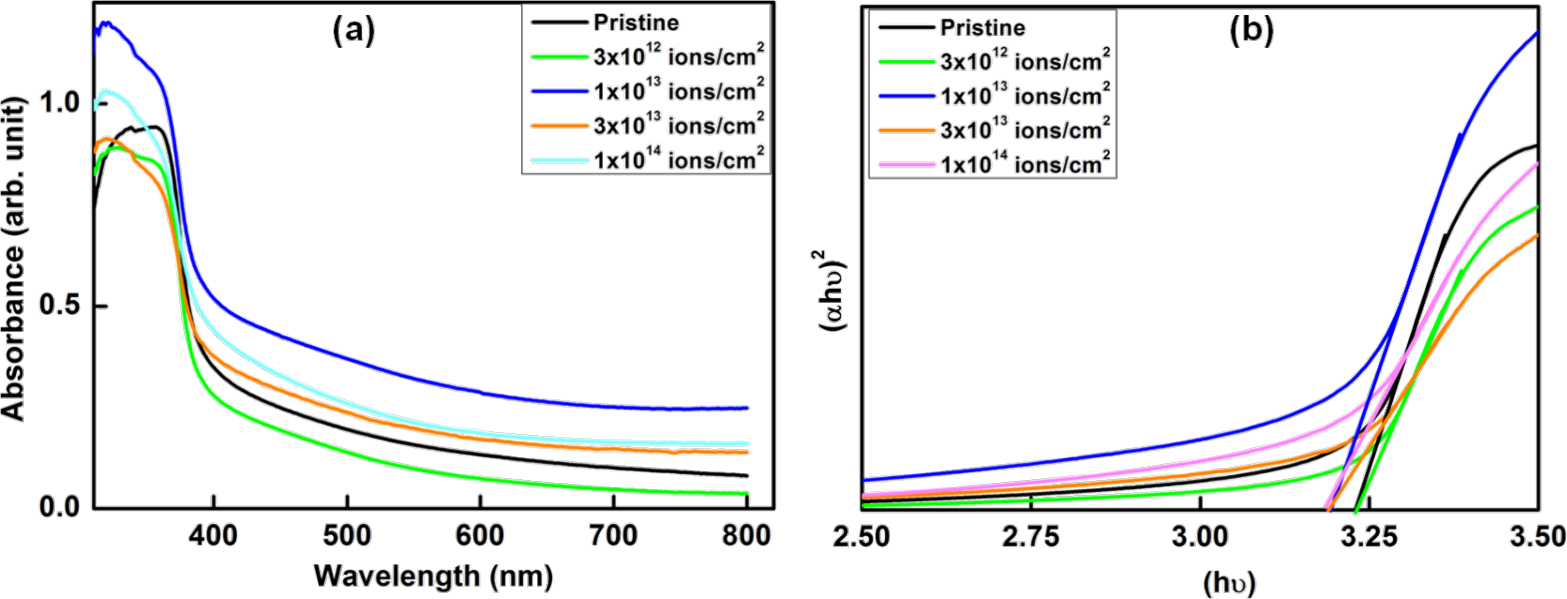
(a) UV–visible absorption spectra of pristine and irradiated ZnO–CuO nanocomposite samples and (b) Tauc plots for estimating the band gap energies.

The UV–visible absorption spectra of the pristine and irradiated nanocomposites are shown in Figure 3a. It can be clearly seen that swift heavy ion irradiation leads to enhanced absorption of the nanocomposite in the visible region. The band gap of pristine nanocomposite and sample irradiated at fluences of 3 × 10^12^, 1 × 10^13^, 3 × 10^13^ and 1 × 10^14^ ions/cm^2^ are estimated from Tauc plots (shown in Figure 3b) to be 3.23, 3.22, 3.19, 3.19 and 3.18 eV, respectively. Swift heavy ion irradiation has been found to result in a decrease in the band gap with an increase in the ion fluence. This can be attributed to doping of Cu into ZnO nanostructures upon swift heavy ion irradiation, which in turn leads to the introduction of defect levels within the band gap. It must be pointed out here that a decrease in the band gap facilitates an easy passage of electrons from the conduction band and therefore leads to an increase in the electron flow in the irradiated samples as compared to the pristine sample. This property can be employed in various optoelectronic devices as well as for achieving improved photocatalytic efficiency.

**Figure 4:**
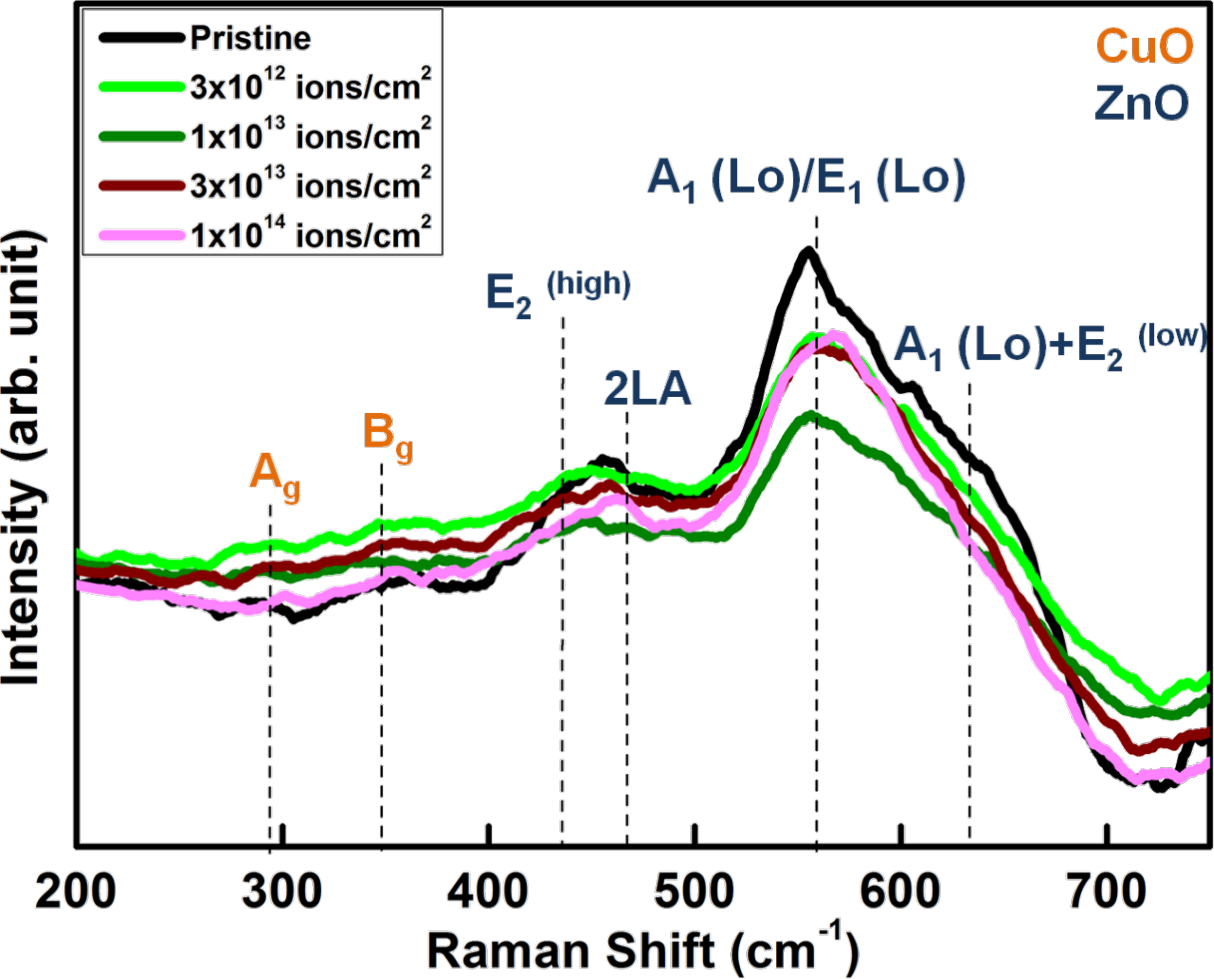
Raman spectra from pristine and irradiated ZnO–CuO nanocomposite samples.

Figure 4 shows the Raman spectra of the pristine and irradiated ZnO–CuO nanocomposites. It is well known that wurtzite ZnO has an irreducible representation *A*_1_ + 2*B*_1_ + *E*_1_ + 2*E*_2_. Since the modes *A*_1_ and *E*_1_ are polar they exhibit two frequencies corresponding to the transverse optical (TO) and the longitudinal optical (LO) phonon modes. The non-polar *E*_2_ mode has two frequencies *E*_2_^(high)^ originating from the motion of oxygen atoms and *E*_2_^(low)^ due to the motion of the Zn sublattice. The Raman spectra of ZnO–CuO nanocomposites exhibit peaks at 283, 352, 435, 468, 560 and 633 cm^−1^. The peaks at 283 and 352 cm^−1^ correspond to the *A*_g_ and *B*_g_ modes of monoclinic CuO respectively, which are due to the vibrations of the oxygen atoms [26]. The peak at 435 cm^−1^ corresponds to the *E*_2_^(high)^ peak characteristic of ZnO and is mainly due to the vibration of the oxygen atoms in the ZnO lattice. The peak at 468 cm^−1^ is attributed to the 2LA mode and the band at 633 cm^−1^ is assigned to the *A*_1_(LO) + E_2_^(low)^ mode of ZnO. The peak at 574 cm^−1^ is due to *A*_1_(LO)/*E*_1_(LO) mode of ZnO [38]. The Raman modes appear slightly shifted due to the presence of stress in the nanocomposites. The co-existence of the Raman modes of both ZnO and CuO also confirms the composite nature of the sample.

**Figure 5:**
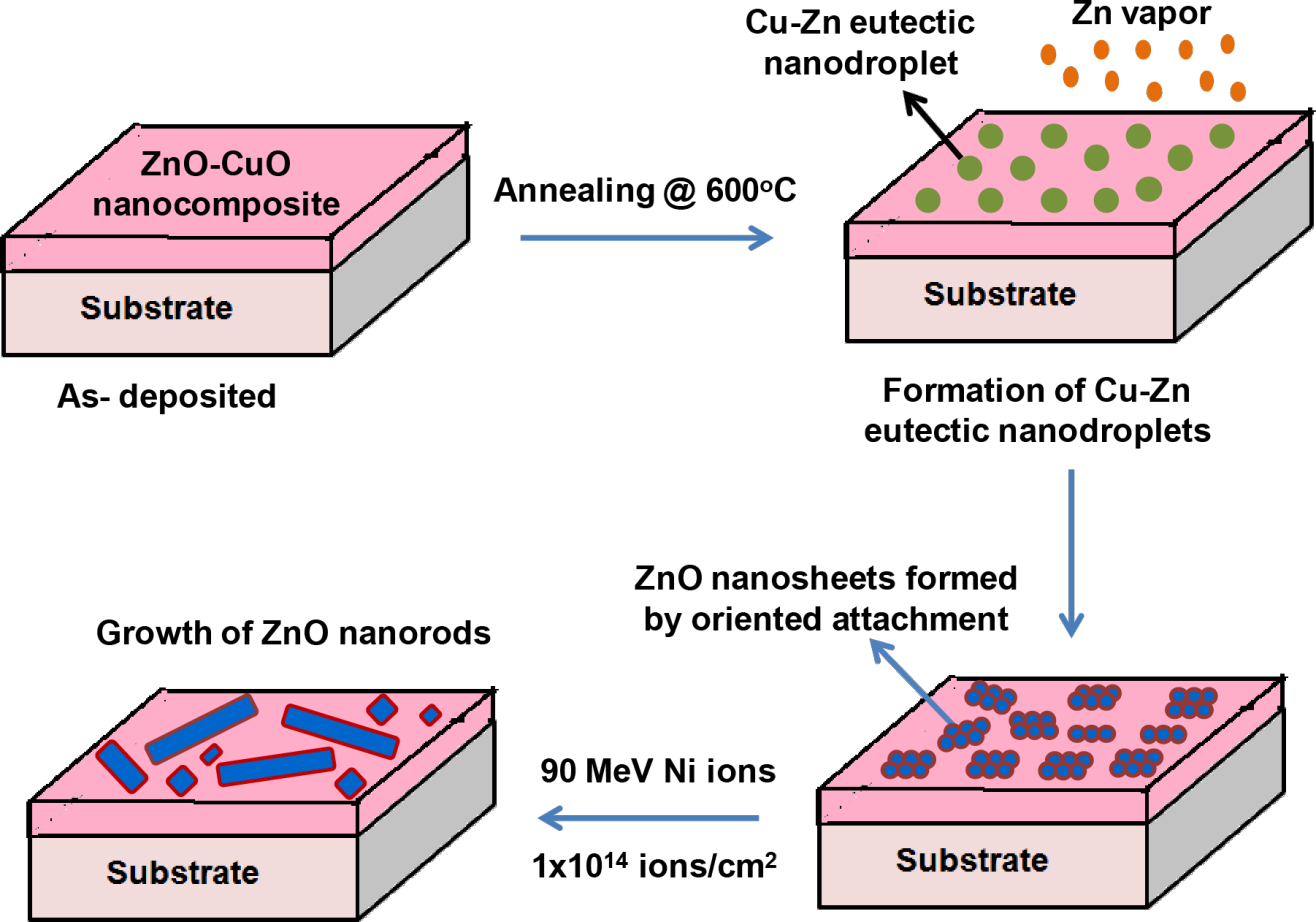
Growth mechanism of ZnO nanorods and nanosheets in the nanocomposites.

Yuan et al. [39] and Thandavan et al. [40] have earlier reported the mechanism of formation of ZnO nanowires mediated by Cu–Zn alloys. The mechanism underlying the growth of ZnO nanosheets and nanorods prepared by carbothermal evaporation of ZnO and Cu followed by annealing can be understood as follows: The carbothermal evaporation of ZnO and Cu mixture led to the deposition of ZnO–CuO nanocomposite film with excess Zn onto the substrate. When this as-deposited film is annealed at 600 °C for 1 h in oxygen atmosphere, it led to the formation of Cu–Zn eutectic nanodroplets at the film surface. Since the melting point of Zn is low (419 °C), during annealing at 600 °C a fraction of the excess Zn atoms evaporates by forming Zn vapor, which dissolves into the Cu–Zn eutectic nanodroplets and oxidizes forming ZnO nanoparticles. These ZnO nanoparticles formed by the catalytic action of Cu–Zn eutectic nanodroplets on the film surface combine through an oriented attachment mechanism, leading to the formation of ZnO nanorods and nanosheets on the surface of the nanocomposites. The schematic diagram depicting the growth mechanism is shown in Figure 5. Irradiation of the ZnO–CuO nanocomposite with 90 MeV Ni^7+^ ions results in localized melting and lateral mass flow leading to the formation of larger nanorod like structures with increased width and distinct facets, as can be seen in Figure 1c and Figure 1d.

**Figure 6:**
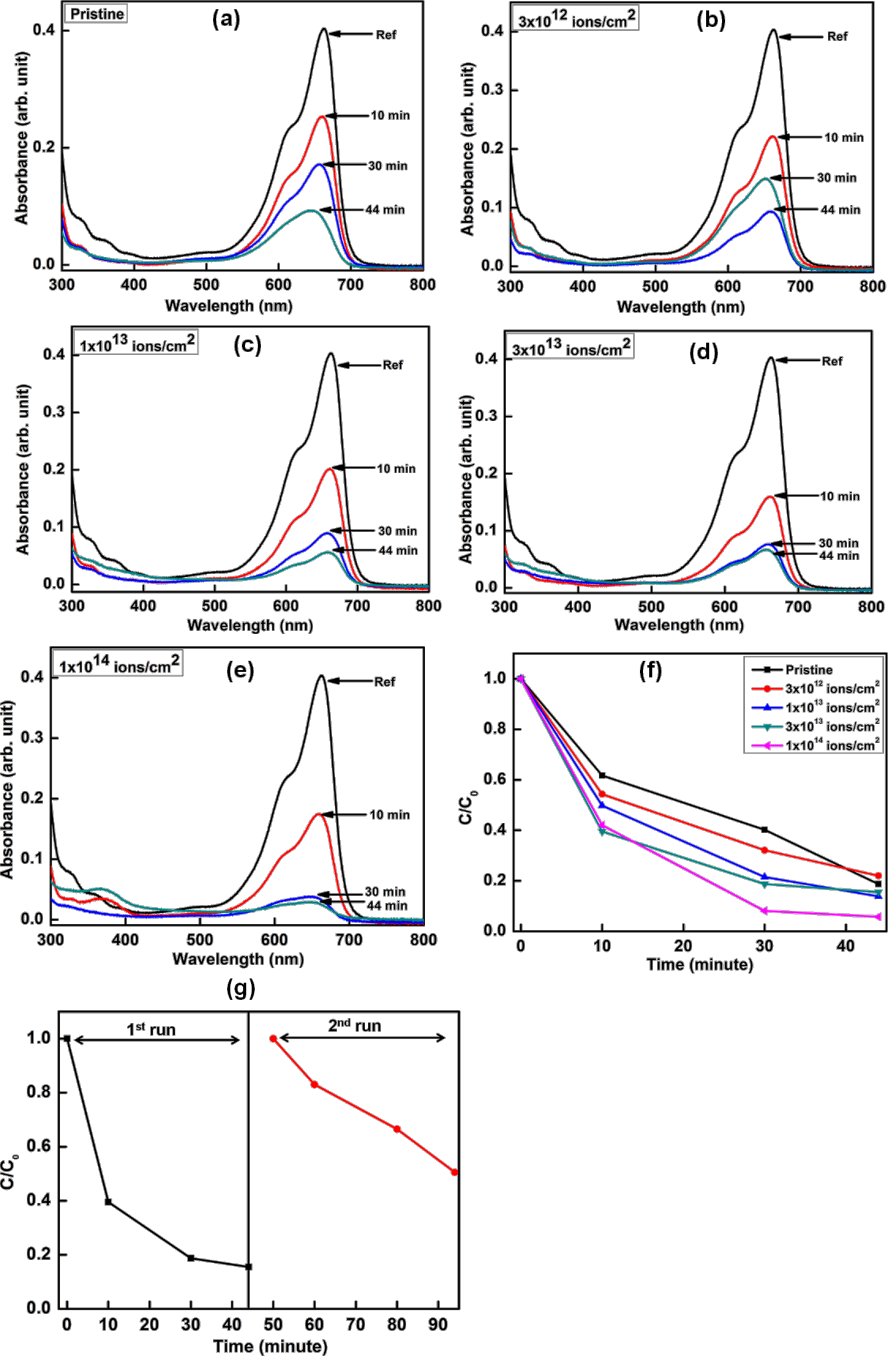
(a–e) UV–visible absorption spectra showing the sun-light-driven time-dependent photocatalytic degradation of MB dye through pristine and irradiated ZnO–CuO nanocomposite samples as photocatalysts. (f) Kinetics of the photocatalytic degradation of MB shown through time dependent variation of *C*/*C*_0_. (g) Repetitive tests using 3 × 10^13^ ions/cm^2^ irradiated sample for two runs of photocatalytic degradation studies on MB dye.

**Figure 7:**
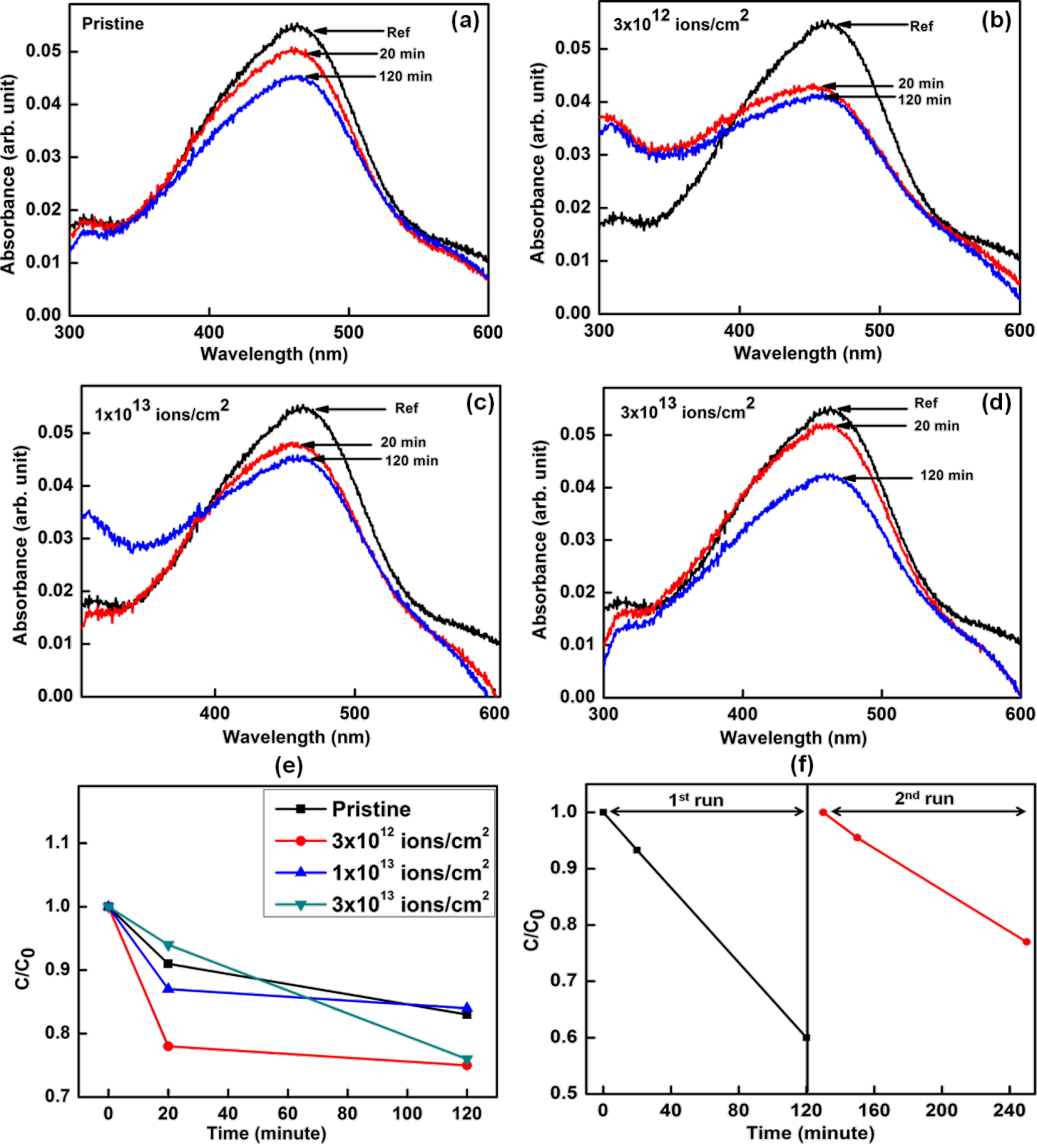
(a–d) UV-visible absorption spectra showing sun light driven time dependent photocatalytic degradation of MO dye using the pristine and irradiated ZnO-CuO nanocomposite samples as photocatalysts. (e) Kinetics of photocatalytic degradation of MO dye shown through time dependent variation of *C*/*C*_0_. (f) Repetitive tests using 3 × 10^13^ ions/cm^2^ irradiated sample for two runs of photocatalytic degradation studies on MO dye.

The photocatalysis studies were carried out by taking MB and MO as model organic dyes to demonstrate the capability of ion beam engineering to optimize the photocatalytic activity of ZnO–CuO nanocomposites. Figure 6 and Figure 7 show the UV–visible absorption spectra of 3.7 μM MB and MO dyes with the pristine and ion-irradiated nanocomposite samples as photocatalysts upon irradiation with sun light for different periods of time. The photocatalytic degradation kinetics was observed by monitoring the characteristic peak of MB at 664 nm and MO at 464 nm as a function of sun light exposure time. It can be clearly seen that the photocatalytic efficiency is highest for the sample irradiated at a fluence of 1 × 10^14^ ions/cm^2^ as compared to the pristine samples and the samples irradiated with lower fluences.

**Figure 8:**
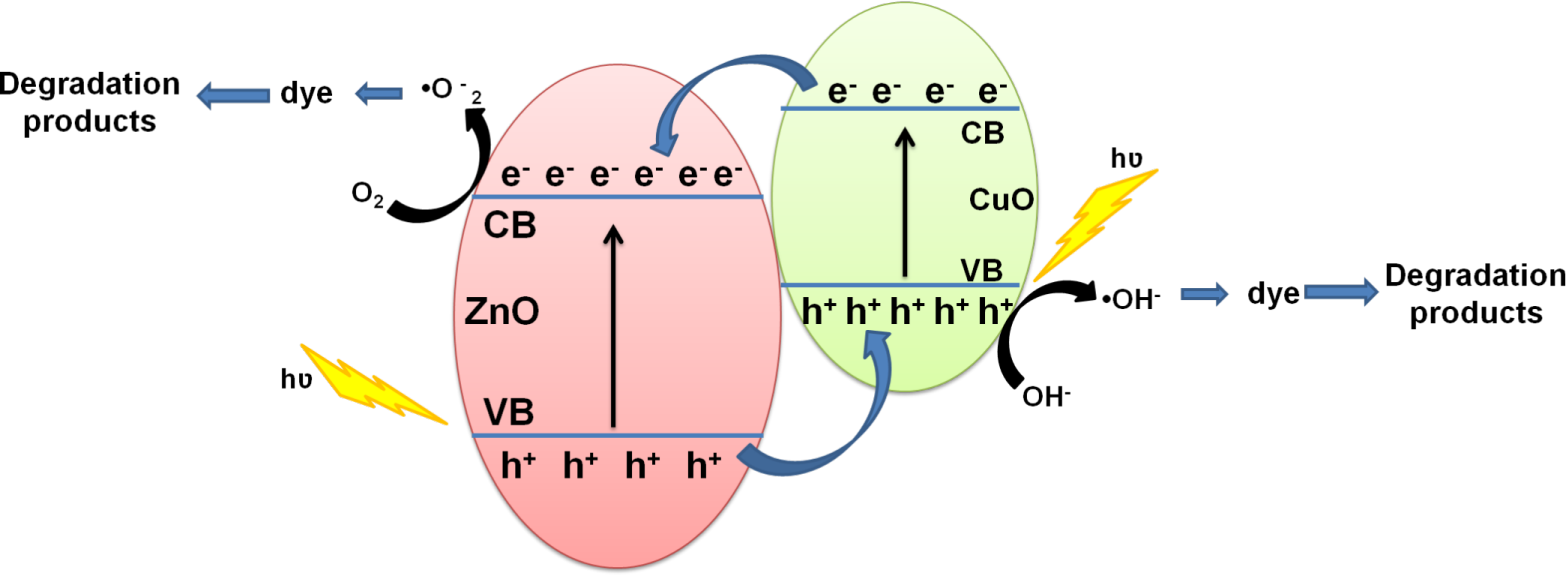
Schematic energy band diagram of ZnO–CuO nanocomposite showing the charge transportation processes leading to sun-light-driven photocatalytic degradation of dye.

The schematic diagram showing the mechanism underlying the photocatalytic degradation of dye through ZnO–CuO nanocomposites is depicted in Figure 8. The mechanism of photocatalysis can be understood as follows. When sun light is incident on ZnO–CuO nano-heterojunctions, electrons from the valance band are excited into the conduction band, leaving behind holes. The photogenerated electrons generated in the conduction band of CuO jump to the conduction band of ZnO, while the holes from the valance band of ZnO transfer to the valance band of CuO. This helps to inhibit the recombination of photogenerated electrons and holes and improves the charge separation efficiency. The oxygen molecules adsorbed on the photocatalyst form superoxide anion radicals (•O_2_^−^) due to their interaction with electrons in the conduction band of ZnO. Surface hydroxyl groups produce highly reactive hydroxyl (•OH) radicals by reacting with holes in the valence band of CuO. The dye molecules are degraded by the reaction with both radicals (•OH and •O_2_^−^) [39,41]. For better understanding of the underlying mechanism, Yang et al. [42–43] have monitored the formation of hydroxyl radicals (•OH) and superoxide radicals (•O_2_^−^) during visible-light-induced photocatalytic degradation of acid orange and 4-nitrophenol. The formation of hydroxyl radicals (•OH) was detected by photoluminescence studies using terephthalic acid as a probe molecule, while 1,4-benzoquinone and ammonium oxalate were used as the quencher of superoxide radicals (•O_2_^−^) and scavenger of holes, respectively. Their studies suggested that hydroxyl radicals (•OH) were not the main active photooxidant and superoxide radicals (•O_2_^−^) formed in the degradation process play the major role in the visible-light-induced photocatalytic degradation. In our study, it can be clearly seen that all the irradiated ZnO–CuO nanocomposites show enhanced photocatalytic activity during the sun light driven degradation of MB and MO dyes.

Our results show that swift heavy ion irradiation leads to significant enhancement in the photocatalytic efficiency of ZnO–CuO nanocomposites toward sun light driven degradation of MB and MO dyes in water. The photocatalytic efficiency increases with increase in ion fluence, reaching the maximum efficiency at the highest fluence of 1 × 10^14^ ions/cm^2^. The enhanced photocatalytic efficiency for the sample irradiated with highest fluence is due to the reduced band gap energy which facilitates the easy transfer of electrons from the valence band to the conduction band. In addition, the improved suppression of recombination of photogenerated charge carriers in ZnO–CuO nanocomposites contributes to the enhanced photocatalytic efficiency. Earlier studies have shown that swift heavy ion irradiation inducing high electronic excitations can be used to control the defect concentration and engineer the shape and size of nanostructured materials [44–45]. Fabricating nanocomposites consisting of 1D and 2D metal-oxide semiconductor nanostructures with higher surface area for efficient adsorption of dye molecules and optimal defect concentration, crystallinity and band gap are important for developing advanced photocatalytic coatings. In this work, we demonstrate that swift heavy ion irradiation can be used to controllably engineer the shape of ZnO nanostructures (nanorods and nanosheets) and enhance the photocatalytic activity of ZnO–CuO nanocomposites, improving their applicability as reusable photocatalysts.

## Conclusion

ZnO–CuO nanocomposite thin films were prepared by carbothermal evaporation of ZnO and Cu, combined with annealing. FESEM studies showed the presence of ZnO nanosheets and nanorods, which are formed by Cu–Zn alloy nanodroplets assisted oriented attachment of ZnO nanoparticles. The effects of swift heavy ion irradiation on the structural, optical, and photocatalytic properties of the nanocomposite were studied. Swift heavy ion irradiation has been found to result in significant enhancement in the photocatalytic efficiency of ZnO–CuO nanocomposites, towards sun light driven degradation of methylene blue and methyl orange dyes in water. The possible mechanism for the enhanced photocatalytic activity of ZnO–CuO nanocomposites is tentatively proposed. We have demonstrated that the combined effects of reduced band gap energy which facilitates easy transfer of electrons from valence band to conduction band, improved sun light utilization and reduced recombination of photogenerated electrons and holes result in the observed enhancement in photocatalytic activity of swift heavy ion irradiated ZnO–CuO nanocomposites.

## Experimental

### Materials

ZnO, Cu and graphite powders were used as the starting materials for the synthesis of ZnO–CuO nanocomposite thin films. ZnO and graphite powders were purchased from Merck, India, while Cu powder was purchased from Loba Chemie. Methylene blue (MB) and methyl orange (MO) were procured from SRL, India. All chemicals used were of analytical grade and were used without any further purification.

### Synthesis and ion beam engineering of ZnO–CuO nanocomposites

ZnO–CuO nanocomposite thin films were synthesized by a simple carbothermal reduction-based vapor deposition process. In this process graphite powder was thoroughly mixed with ZnO powder in a ratio of 1:10 along with 30% of Cu metal powder and pelletized. Thin films on thoroughly cleaned silica glass substrates were deposited by thermal evaporation under a vacuum of 1.4 × 10^−5^ Torr. The as-prepared samples were then annealed at 600 °C for 1 h in oxygen flow. These samples were then irradiated with 90 MeV Ni^7+^ ions to fluences varying from 3 × 10^12^ to 1 × 10^14^ ions/cm^2^.

### Characterization of ZnO–CuO nanocomposites

The structural and optical properties of the as-prepared and irradiated samples were characterized by X-ray diffraction (XRD), field emission scanning electron microscopy (FESEM), Raman spectroscopy and UV–visible absorption spectroscopy. Grazing incidence XRD patterns were recorded by Bruker D8 Advance diffractometer at grazing incidence of 2° using Cu Kα (λ = 1.5406 Å) source operating at 40 kV and 40 mA. Raman spectra were recorded by using a Horiba Jobin Yvon LabRam with a spot size of 1 µm at a wavelength of 488 nm.

### Photocatalytic measurements

The photocatalytic activity of the pristine and irradiated samples was evaluated by monitoring the degradation of methylene blue (MB) and methyl orange (MO) dyes in water under sun light irradiation in a similar manner, as described previously in [41]. For the photocatalytic studies, aqueous solutions of 3.7 μM MB and 3.7 μM MO with the pristine and irradiated samples dipped in them were irradiated with sun light for different durations of time (10, 30 and 44 min). These experiments were carried out at mid day during peak summer at the same time spanning up to 44 min to ensure exposure with sun light of maximum luminosity. Following sun light irradiation, the photocatalysts were removed from the aqueous dye solutions. The concentrations of the dye in the resultant solutions were monitored by UV–visible absorption spectroscopy with double distilled water as the reference medium, in the wavelength range of 300–800 nm.

## References

[1] Park J-H, Choi Y-J, Park J-G (2005). J Cryst Growth.

[2] Chen S J, Liu Y C, Shao C L, Mu R, Lu Y M, Zhang J Y, Shen D Z, Fan X W (2005). Adv Mater.

[3] Xu C X, Sun X W, Dong Z L, Yu M B (2004). Appl Phys Lett.

[4] Umar A, Hahn Y B (2006). Nanotechnology.

[5] Cao B, Cai W, Li Y, Sun F, Zhang L (2005). Nanotechnology.

[6] Mishra Y K, Kaps S, Schuchardt A, Paulowicz I, Jin X, Gedamu D, Freitag S, Claus M, Wille S, Kovalev A (2013). Part Part Syst Charact.

[7] Mishra Y K, Kaps S, Schuchardt A, Paulowicz I, Jin X, Gedamu D, Wille S, Lupan O, Adelung R (2014). KONA.

[8] Bhardwaj N, Kuriakose S, Pandey A, Sharma R C, Avasthi D K, Mohapatra S (2014). J Alloys Compd.

[9] Jain A, Panwar S, Kang T W, Kumar S (2013). J Mater Sci: Mater Electron.

[10] Yang N, Yang H, Qu Y, Fan Y, Chang L, Zhu H, Li M, Zou G (2006). Mater Res Bull.

[11] Dong H, Liu Y, Lu J, Chen Z, Wang J, Zhang L (2013). J Mater Chem C.

[12] Ko S H, Lee D, Kang H W, Nam K H, Yeo J Y, Hong S J, Grigoropoulos C P, Sung H J (2011). Nano Lett.

[13] Zhang Q, Dandeneau C S, Zhou X, Cao G (2009). Adv Mater.

[14] Kim I-D, Homg J-M, Lee B H, Kim D Y, Jeon E-K, Choi D-K, Yang D-J (2007). Appl Phys Lett.

[15] Cheng X L, Zhao H, Huo L H, Gao S, Zhao J G (2004). Sens Actuators, B.

[16] Xu H, Liu X, Cui D, Li M, Jiang M (2006). Sens Actuators, B.

[17] Gedamu D, Paulowicz I, Kaps S, Lupan O, Wille S, Haidarschin G, Mishra Y K, Adelung R (2014). Adv Mater.

[18] Zimmler M A, Bao J, Capasso F, Muller S, Ronning C (2008). Appl Phys Lett.

[19] Pivin J C, Socol G, Mihailescu I, Berthet P, Singh F, Patel M K, Vincent L (2008). Thin Solid Films.

[20] Lee S-H, Han S-H, Jung H S, Shin H, Lee J, Noh J-H, Lee S, Cho I-S, Lee J-K, Kim J (2010). J Phys Chem C.

[21] Grätzel M (2004). J Photochem Photobiol, A.

[22] Yang K, She G-W, Wang H, Ou X-M, Zhang X-H, Lee C-S, Lee S-T (2009). J Phys Chem C.

[23] Zeng H, Cai W, Liu P, Xu X, Zhou H, Klingshirn C, Kalt H (2008). ACS Nano.

[24] Xu F, Shen Y, Sun L, Zeng H, Lu Y (2011). Nanoscale.

[25] Reimer T, Paulowicz I, Roder R, Kaps S, Lupan O, Chemnitz S, Benecke W, Ronning C, Adelung R, Mishra Y K (2014). ACS Appl Mater Interfaces.

[26] Wang W, Zhou Q, Fei X, He Y, Zhang P, Zhang G, Peng L, Xie W (2010). CrystEngComm.

[27] Borgohain K, Mahamuni S (2002). J Mater Res.

[28] Kidowaki H, Oku T, Akiyama T, Suzuki A, Jeyadevan B, Cuya J (2012). J Mater Sci Res.

[29] Waser O, Hess M, Güntner A, Novák P, Pratsinis S E (2013). J Power Sources.

[30] Saravanan R, Karthikeyan S, Gupta V K, Sekaran G, Narayanan V, Stephen A (2013). Mater Sci Eng, C.

[31] Simon Q, Barreca D, Gasparotto A, Maccato C, Tondello E, Sada C, Comini E, Sberveglieri G, Banerjee M, Xu K (2012). ChemPhysChem.

[32] Habibi M H, Karimi B, Zendehdel M, Habibi M (2013). Spectrochim Acta, Part A.

[33] Mishra Y K, Avasthi D K, Kulriya P K, Singh F, Kabiraj D, Tripathi A, Pivin J C, Bayer I S, Biswas A (2007). Appl Phys Lett.

[34] Amekura H, Okubo N, Ishikawa N, Tsuya D, Mitsuishi K, Nakayama Y, Singh U B, Khan S A, Mohapatra S, Avasthi D K (2013). Appl Phys Lett.

[35] Kumar S, Kumar R, Singh D P (2009). Appl Surf Sci.

[36] Kumar Y, Herrera-Zaldivar M, Olive-Méndez S F, Singh F, Mathew X, Agarwal V (2012). Nanoscale Res Lett.

[37] Agarwal D C, Kumar A, Khan S A, Kabiraj D, Singh F, Tripathi A, Pivin J C, Chauhan R S, Avasthi D K (2006). Nucl Instrum Methods Phys Res, Sect B.

[38] Kuriakose S, Satpati B, Mohapatra S (2014). Phys Chem Chem Phys.

[39] Yuan L, Wang C, Cai R, Wang Y, Zhou G (2013). J Appl Phys.

[40] Thandavan T M K, Gani S M A, Wong C S, Md Nor R (2014). J Nanomater.

[41] Kuriakose S, Bhardwaj N, Singh J, Satpati B, Mohapatra S (2013). Beilstein J Nanotechnol.

[42] Yang Y, Zhang G, Xu W (2012). J Colloid Interface Sci.

[43] Yang Y, Zhang G, Yu S, Shen X (2010). Chem Eng J.

[44] Ghosh S, Ganesan V, Khan S A, Ayyub P, Kumar N (2006). Appl Surf Sci.

[45] Shirai M, Tsumori K, Kutsuwada M, Yasuda K, Matsumura S (2009). Nucl Instrum Methods Phys Res, Sect B.

